# Development of a toolkit for educators of the wheelchair service provision process: the Seating and Mobility Academic Resource Toolkit (SMART)

**DOI:** 10.1186/s12960-020-0453-6

**Published:** 2020-02-18

**Authors:** Paula W. Rushton, Karen Fung, Mélina Gauthier, Mary Goldberg, Maria Toro, Nicky Seymour, Jon Pearlman

**Affiliations:** 1 School of Rehabilitation, University of Montréal, Montréal, Canada; 2 CHU Sainte-Justine Research Centre, 5200 rue Bélanger, Montréal, QC H1T 1C9 Canada; 3 Rehabilitation Science and Technology, University of Pittsburgh, Pittsburgh, USA; 4 Human Engineering Research Laboratories, University of Pittsburgh, Pittsburgh, USA; 5 Department of Physical Therapy, Universidad CES, Medellín, Colombia; 6 Motivation Charitable Trust, Cape Town, South Africa

**Keywords:** Wheelchair service provision, Wheelchair education, Integration, Curricula, Formal rehabilitation education programs

## Abstract

**Background:**

Insufficient wheelchair training among rehabilitation professionals has been identified as an important factor that hinders access to appropriate wheelchair services. The aim of this study was to develop a toolkit to promote the integration of wheelchair education into academic curricula of rehabilitation programs.

**Methods:**

A participatory action research design was carried out in three phases: (1) development of the Initial and Alpha Versions involving secondary analyses of surveys (*n* = 72), interviews (*n* = 14), and academic training partners meeting presentations (*n* = 16); (2) development of the Beta Version based on feedback from collaborators (*n* = 21); and (3) development of the Launch Version based on feedback from participants attending presentations of the Beta Version at conferences, symposiums, and webinars (*n* = 94).

**Results:**

Over 100 individuals participated in reviews of the *Seating and Mobility Academic Resource Toolkit* (SMART). Initial development addressed modifiable factors that perpetuate insufficient wheelchair education in academic curricula (e.g., limited awareness, limited expertise). Internal feedback on the web-based Alpha Version resulted in modifications of appearance and multimedia, structure and design, and navigation. External feedback then led primarily to fine-tuning the navigation of SMART. Positive reviews were received from global wheelchair professionals (i.e., educators, researchers, clinicians). The Launch Version of the SMART (smart.wheelchairnetwork.org) provides a forum for sharing and accessing resources to inform the integration and enhancement of wheelchair content into university rehabilitation programs.

**Conclusions:**

As an open-source open-access online “living document,” SMART has the potential to promote the integration of wheelchair service provision education into academic curricula of rehabilitation programs. Future studies will explore the ease of use and the effectiveness of the SMART.

## Introduction

Personal mobility is a fundamental and basic human right [1]. Insufficient wheelchair service provision violates the United Nations Convention on the Rights of Persons with Disabilities (Article 20) [1]. Not only does this issue affect the 75 million people worldwide who require a wheelchair for mobility, its negative consequences are numerous, including compromised physical health, safety, quality of life, vocational status, and educational status [2, 3]. While the reasons for insufficient wheelchair provision are multifaceted, a factor of increasingly accepted importance is the shortage of competent professionals [2].

The need to build a competent workforce for the provision of assistive technology is embedded within the World Health Organization (WHO)’s Global Cooperation on Assistive Technology (GATE) five interlinked areas (i.e., people, policy, products, provision, and personnel) highlighting its universal importance [4]. Indeed, it was included as a priority theme in the Global Priority Research Agenda [5] and was integrated as a topic in the first Global Research, Innovation and Education on Assistive Technology (GREAT) Summit [6, 7]. The need for a systematic approach to personnel education and training was identified at the GREAT Summit and in its resultant position papers [6, 8]. Further support for this need is captured by the sector goal developed at the 2018 Wheelchair Stakeholders’ Meeting which states “By 2023, 10 countries will have new or strengthened evidence-based, adequately resourced, integrated wheelchair services supported by policies, competent personnel and a range of appropriate wheelchairs.” One of the top five integrated priority action items related to this sector goal is to *support competency development* by developing a competent workforce of multi-sectoral wheelchair service personnel through the use of regional training centers that utilize existing and newly developed resources [9].

Current approaches to providing education and training to wheelchair service providers include formal and informal methods by several different entities, including universities, non-governmental organizations, and humanitarian organizations [10]. The recently proposed systematic approach to training assistive technology personnel identified the need for basic competence across a range of devices at the community level, followed by specialization of personnel in product groups, including personal mobility devices such as wheelchairs [11]. This method aligns with both the Wheelchair Service Provider Certification process offered by the International Society of Wheelchair Professionals (ISWP) [12], as well as evolving health care professional education standards [13]. As various approaches to education and training, as well as evaluation of competency are studied and evolve, the variability in the content and curricula that is taught, the methods and pedagogic approaches used to teach the material, and how knowledge and level of competency is evaluated has become apparent [10, 14–17].

Although ideally situated to provide education and training to current and emerging wheelchair service providers [11], universities face certain challenges in integrating wheelchair education into curricula. Educators in university rehabilitation programs have identified issues at three of the five states of the integration process: advocacy (e.g., limited faculty interest or resistance to integration of wheelchair content), planning (e.g., lack of awareness of resources), and course development and delivery (e.g., lack of time, lack of expertise) [18]. Even in the remaining two states of integration, i.e., first-time implementation and improvement, both the value of and the need for resources have been identified as a means of overcoming these challenges [14, 18]. Recognizing the variety of challenges that exist, yet also the variety of expertise and resources that are available globally, the objective of this study, conducted by the ISWP, was to develop a *Seating and Mobility Academic Resource Toolkit* (SMART) [19]. The goal of this dynamic toolkit is to provide educators with a means of sharing and accessing resources that will inform the integration and enhancement of wheelchair content into university rehabilitation programs and ultimately contribute to the development of a competent workforce of wheelchair service personnel.

## Methodology

### Study design

**Fig. 1:**
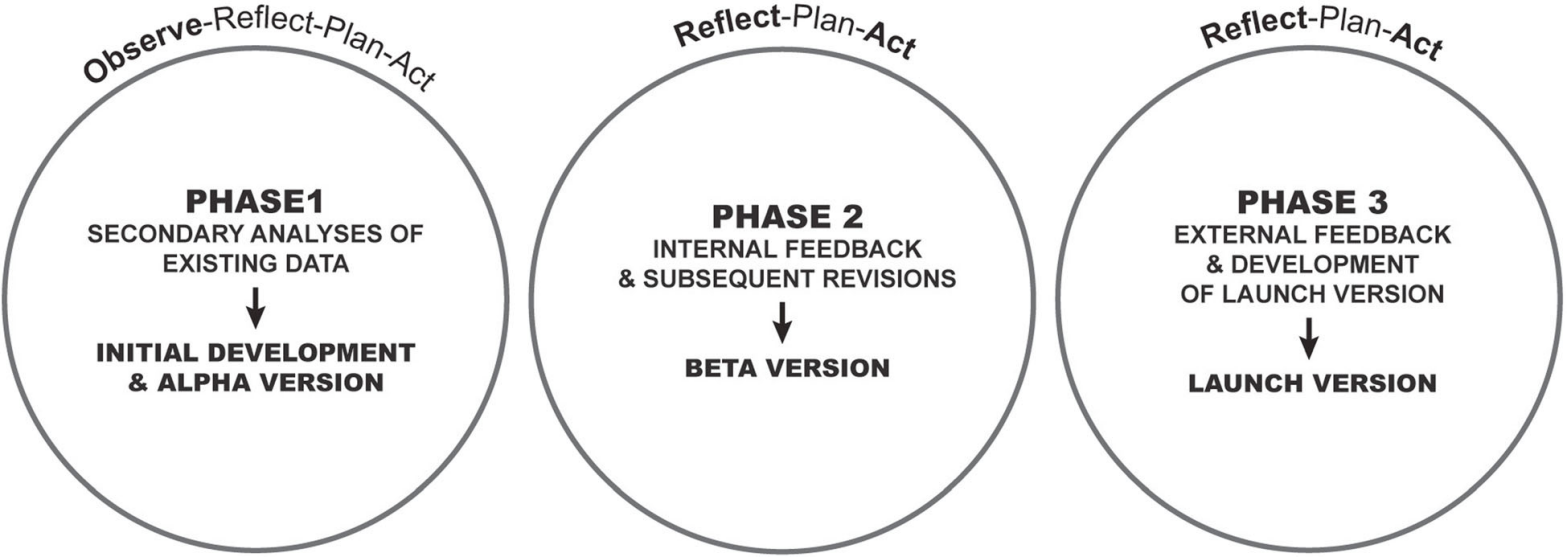
SMART development process. Cycles of participatory action research (PAR) activities occurred throughout the three phases of the SMART development process. In bold are PAR activities which were emphasized. Arrows point to the product of each phase

To develop the SMART, this study employed a participatory action research (PAR) approach, with its cyclical activities of Observe, Reflect, Plan and Act [20]. Led by an ISWP Task Force of individuals who recognized the need for sharing resources among educators of wheelchair service provision (described below), this study involved three phases (Fig. 1) that occurred during an 18-month period in 2017–2018. Embedded within these phases were the uses of multiple methods and data sources (Table 1). Given their nature, each phase received emphasis on specific activities of the PAR approach (Fig. 1), with the Observe activities occurring only in Phase 1. Project meetings in all phases were held via Adobe Connect and audio recorded with the exception of sessions conducted at conferences.

**Table 1:** Methodology based on participatory action research approach

	Phase 1 Secondary analyses of existing data	Phase 2 Internal feedback and subsequent revisions	Phase 3 External feedback and development of Launch Version
Participants	SMART Task Force	● ISWP Training Working Group ● ISWP Integration Sub-committee ● Academic Training Partners	Attendees at 3 international conferences and 3 SMART informational webinars
Observe	Secondary analyses of existing data (survey and interviews)	–	–
Reflect	● Sharing secondary analyses results with Integration Sub-committee ● Creation of Initial Development	Feedback sessions	Feedback sessions
Plan	Determination of the platform design and its content by SMART Task Force	Review of suggestions based on resources and feasibility by SMART Task Force	Review of suggestions based on resources and feasibility by SMART Task Force
Act	● Development of Alpha Version	● Modifications made to Alpha Version ● Development of Beta Version	● Modifications made to Beta Version ● Development of Launch Version

### Participants

#### SMART Task Force

Responsible for all three phases of toolkit development, including all data analyses, was the SMART Task Force, which included an occupational therapist with a PhD in Rehabilitation Sciences who is a faculty member at a Canadian university, an educator with a PhD in Administrative and Policy Studies of Education who is a faculty member at an American university, an occupational therapist who has a Masters in Human Rehabilitation and Interdisciplinary Health Studies and is currently practicing in South Africa, a biomedical engineer with a PhD in Rehabilitation Sciences who is a faculty member at a Colombian university, a physical therapist who is a consultant for international disability programs, a Rehabilitation Science and Technology student at an American university, and an occupational therapy student at a Canadian university. The SMART Task Force members were a sub-set of members the ISWP groups described below who were interested and willing to dedicate the time to lead the development of SMART.

#### Internal participants

Three multidisciplinary, internationally representative ISWP groups were involved as internal participants, including the Training Working Group (a group dedicated to improving the wheelchair provision process through competent training and service delivery), the Integration Sub-Committee (a committee of the Training Working Group, whose aim is to promote the integration of wheelchair service provision education into the curricula of rehabilitation professional education programs), and the Academic Training Partners Group (a by-product of the Integration Sub-Committee that consists of educators of the wheelchair service provision process from universities and training facilities worldwide who meet virtually to share information and resources regarding their education programs and learn from others). As described above, the mandate and membership of each group is slightly different and there is minimal overlap in membership between each group. To obtain sufficient and diverse feedback, we included in the SMART development process all three ISWP groups whose mandate included improving the wheelchair provision process through improved education. The role of each groups was the same. In Phase 1, 12 internal participants were involved, which included 11 from high-resourced settings [HRS] and 1 from a low-resourced setting [LRS], 11 females and 1 male, 4 graduate students, 4 university professors, 3 administrative members, and 1 service development manager for a non-profit organization. In Phase 2, there were 9 internal members involved, including 7 from HRS and 2 from middle-resourced settings [MRS], 8 females and 1 male of which 5 were university professors, 3 administrative members, and 1 a graduate student.

#### External participants

Thirty people attended the SMART presentation at the World Federation of Occupational Therapists Congress (WFOT) 2018 and 11 people (10 from HRS and 1 from MRS) attended the SMART Workshop at the Rehabilitation Engineering and Assistive Technology Society of North America (RESNA) 2018 conference. At the European Seating Symposium (ESS) SMART presentation, 18 attendees registered their geographic and professional affiliations (14 from HRS, 3 from MRS, and 1 from LRS). Of these attendees, 6 reported working in a university and 7 in a non-educational institution, which included rehabilitation centers (*n* = 4). There was a total of 35 participants at the English (*n* = 25), Spanish (*n* = 9), and French (*n* = 1) SMART webinars.

### Phase 1

The methods in the Observe Phase of the PAR approach involved a secondary data analysis of existing data [21] (see Table 1). These data were from a survey study and a semi-structured interview study conducted to develop an enhanced understanding of the current wheelchair service provision education provided in professional rehabilitation programs worldwide [14, 18]. The secondary data analysis plan involved a deductive qualitative approach whereby a set of reflective questions were used to conduct an in-depth analysis of the barriers to integration of wheelchair content into curricula and how these barriers could be overcome through the use of an online SMART using facilitators identified from the existing data as well as new strategies. NVivo software (Version 11.4.1, QSR International Pty Ltd, Victoria, Australia) was used to organize the data.

Findings from the secondary data analysis were represented through a force field analysis graph [22], whereby the factors perpetuating and attenuating the problem of insufficient wheelchair education in academic curricula were explored for their commonality, importance, and potential for change. Each factor was given a value (i.e., + 5 to − 5) to reflect how frequently it was identified by individual participants and its participant-perceived impact. Based on technical and economic feasibility of potential toolkit features, the Task Force judged whether or not each factor was considered modifiable by SMART.

In Reflect, the SMART Task Force reviewed the data analysis findings to initiate SMART development. Next, in a 1-h meeting, the Task Force shared the findings and initial thoughts regarding the SMART development with the ISWP Integration Sub-Committee. To gather resources pertaining to wheelchair education for the SMART, invitations to contribute educational resources were then extended to the Integration Sub-Committee and Academic Training Partners group. For the Plan and Act activities, Zoltun Design (Pittsburgh, USA), a graphic design company, was hired to develop the Alpha Version of the SMART website. Debuting as an English website, the objective of SMART was to encourage more educators to share and promote wheelchair education resources to increase and enhance wheelchair service provision training worldwide.

### Phase 2

During Reflect, a 1-h meeting was held with the ISWP Training Working Group, Integration Sub-committee, and Academic Training Partners whereby a short PowerPoint presentation on the development of SMART was shared. During the session, whereby all feedback was welcomed, overall general impressions were requested regarding website (a) appearance and multimedia, (b) structure and design, (c) navigation, and (d) content. During Plan and Act, the SMART Task Force Team prioritized and readily adopted all suggestions that were technically and economically feasible. In close collaboration with the website design company, required changes were made.

### Phase 3

In Reflect, to obtain external feedback from the global wheelchair professional community (i.e., educators, researchers and clinicians), the SMART Task Force presented the SMART at three international conferences in 2018 (i.e., WFOT, ESS, RESNA) and held three SMART Informational Webinars (English, French, and Spanish). The presentations and webinars provided a SMART overview (i.e., its purpose, development process, intended users, and a demonstration of the various menu options with a focus on the resources available), and interactive discussions were facilitated. The opportunity to provide feedback via an online discussion board was also provided. When possible, information on the location and organization were collected from attendees. The same methods from Phase 2 were used in Plan and Act. Google Analytics (Mountain View, USA) was used to obtain data on the SMART website traffic.

## Results

### Phase 1

**Fig. 2:**
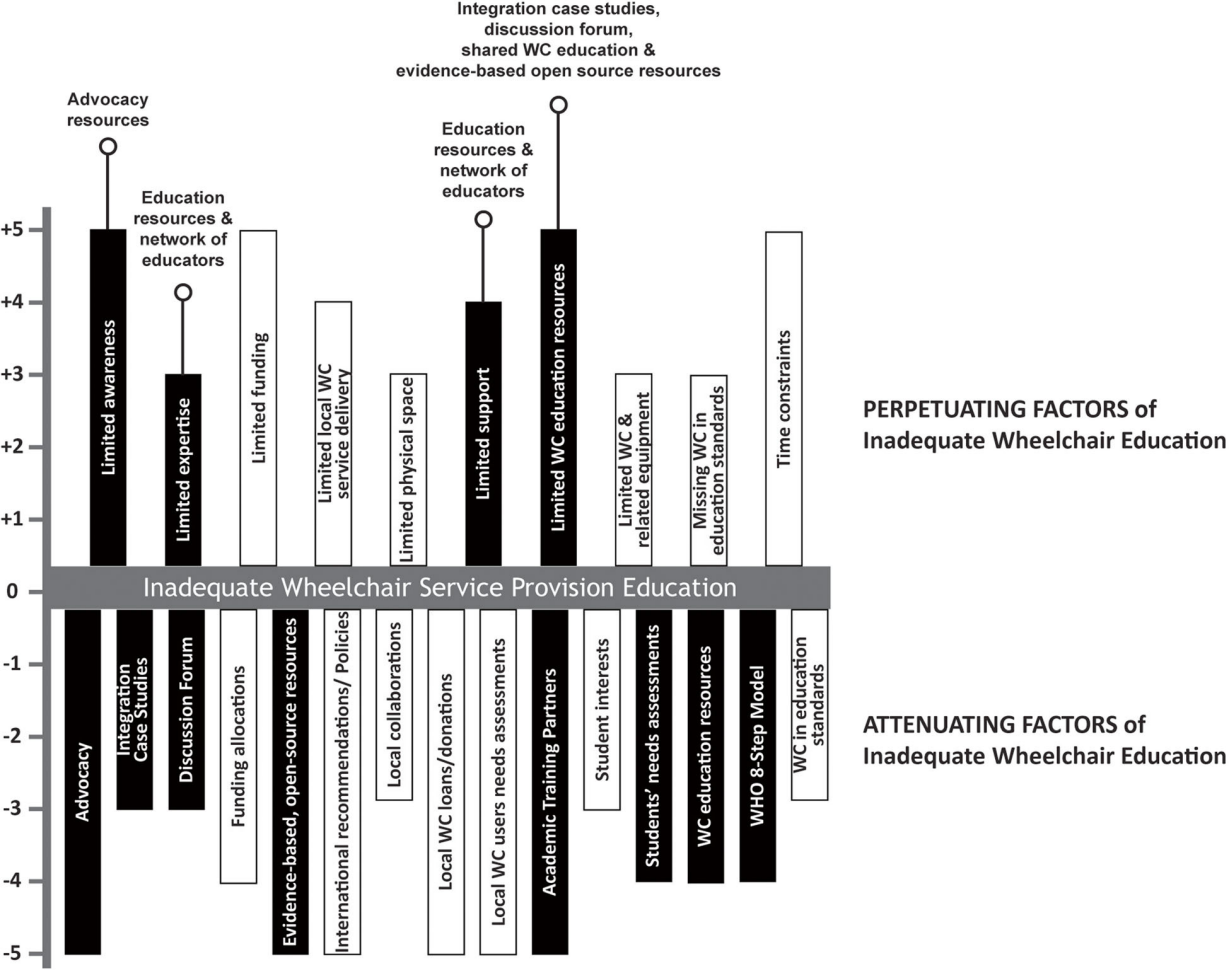
Force field chart of factors associated with the problem of insufficient wheelchair service provision education in academic professional rehabilitation programs. In their respective category (attenuating or perpetuating), factors are listed in alphabetical order. Black bars represent modifiable factors to be addressed by SMART. White bars represent factors not addressed in SMART. Open circles indicate SMART features that address the associated factors. WC: wheelchair

The results from the secondary analysis of existing data yielded a force field diagram presenting factors perpetuating and attenuating the problem of insufficient wheelchair education in academic curricula (Fig. 2). The black bars in the figure represent modifiable factors to be addressed by SMART. The white bars represent factors not addressed in SMART. The open circles indicate SMART features that address the associated factors.

The initial development of SMART involved determination of the platform, layout, menu, and content type. An online platform was chosen to maximize accessibility for all users, to facilitate a high volume of resources to be uploaded by users, then managed and shared by the website administrator and to enable the constant evolution of the toolkit. Resources shared by members of the Task Force were included in the initial version and organized according to the WHO 8-step Wheelchair Service Provision Model [2]. The online platform was developed by the design company.

Table 2 details the feedback and modifications that led from the Initial to Alpha to Beta to Launch versions of SMART. The development of the SMART Alpha Version from its initial development focused mainly on factors related to appearance and multimedia (e.g., visual representation of SMART target audience), structure and design (e.g., efficient method for sharing of resources), navigation (e.g., search filters to access resources), and content (e.g., development of Integration Snapshots, addition of ISWP and Integration Sub-Committee information).

**Table 2:** Development process of SMART versions based on feedback received

Feedback Received (Reflect)	Modification (Act)
Appearance and multimedia	
Better visually represent the impact that SMART hopes to achieve ^i^	Homepage: photographs of diverse groups of students ^α^
Facilitate the use of SMART for visually impaired users ^β^	Toggles onto the side of webpage to enable larger fonts and/or higher color contrasts ^L^
Highlight new SMART features and enhancements ^β^	Homepage: widget “Recent News and Updates” with infographics ^L^
Structure and design	
Better explain the role of ISWP ^i^	“About us” webpage ^α^
How can users to upload resources ^i^	“Contributions” webpage with dropbox ^α^
How can educators help indicate the relevancy of their resources ^β^	“Contribute Form” and description of proper filename nomenclature ^L^
Receive feedback/questions on SMART from users ^α^	Homepage: Email link to ISWP, analytics were made accessible to administrators ^β^
Explain the purpose, intended users, development process and future developments of SMART ^α^	“About the Toolkit” ^β^
How can users quickly contact ISWP for more information ^α^	“Contact Us” button on all webpages ^β^
Need to organize resources uploaded onto SMART website ^α^	Nomenclature developed for files uploaded to SMART ^β^
Invite users to participate in ISWP integration activities ^α^	“Participate/Get Involved” button ^β^
Where to store occupational therapy [and other programs] education standards and related links ^α^	“Advocacy” folder ^β^
Include resources aimed towards community-based facilities in addition to materials geared towards educational institutions ^α^	Under the “Advocacy” folder, sub-folder for “Community-based Services and/or Activities” ^β^
Include resources containing additional information (e.g., student evaluation rubrics) ^α^	Under the “Syllabus” folder, sub-folder for Course Specifications ^β^
Foster interaction and collaborations within the global community of wheelchair educators ^α^	Discussion board, “Interactive Map” with users who wish to be featured their contact information and their integration story ^β^
Generate more interactions in the discussion board ^β^	Continuous revision at least once a week, periodically forum topics initiation, possibility for all users to start a new topic ^L^
Concerns on the lack of vetting measures for resources uploaded to SMART, the ownership of the resources uploaded to SMART ^β^	“Conditions of Use” to inform users of specific conditions related to copyright, acknowledgement of original source and translation of materials into different languages ^L^
Navigation	
Organize search of resources on SMART website ^i, α, β^	Search filters by • Educational institutions ^α^ • Document formats (e.g., text documents, images, videos) ^α^ • WHO 8-Step Wheelchair Service Provision Model ^β^ • International Classification of Functioning, Disability and Health framework ^β^ • Integration States ^β^ • Resource settings (low, middle, high) ^L^ • Languages ^L^ • Rehabilitation Professions ^L^ • Content types (e.g., case studies, lab guides, online modules) ^L^
Lack of uniformity in its navigation ^β^	SMART menu at the top of each webpage for ease of navigation ^L^
Content	
With the established nomenclature, file names are very long. ^α^	Acronyms in SMART were considered inappropriate, as they may not translate well in different languages. “Contribution Form” helps users identify elements of their resources. ^β^
Need to inform how the resources are up-to-date ^α^	All files are dated. ^β^
Keep the shared resources as current as possible ^α^	Rather than document files of shared resources, website links are provided to direct users to original online sources. ^β^
Need information about the integration of wheelchair education into academic curricula ^i^	Inclusion of results from survey study, interview study with links to other scientific articles on the topic and academic training partners (past and upcoming activities) ^α^
Other wheelchair education resources other than ones shared and/or created by other educators ^α^	List of useful wheelchair education related textbooks and other publications ^β^
How can users connect with the authors of the shared resources to receive more information ^β^	Contact information requested on all SMART resources ^L^
How can users be sure whether or not they can use the shared resources? ^i^	Authors’ permission requested in the “Contribution Form” unless the tools are open-source. ^α^
Text-based case studies are cumbersome to read. ^i^	Text modified into visual representations – “Integration Snapshots.” ^α^

^i^Initial development; ^α^Alpha Version; ^β^Beta Version; ^L^Launch Version

### Phase 2

Based on the feedback session with the ISWP Training Working Group, Integration Sub-committee, and Academic Training Partners, important changes were made to the structure and design of the Alpha Version of SMART. New sections were added in order to better organize the received resources according to the integration states (advocacy, planning, course development and delivery, first-time implementation, and improvement). When possible, website links were provided for web-based resources to ensure that SMART contained the most updated versions of wheelchair education resources. New extensive search filters now provided specifics on the individual user and their academic situation, rather than only details about the resources themselves. The main advantage to the availability of extensive search filters was that all resources relevant to the specific user will be proposed whether or not the user is aware of and actively searching such resources.

### Phase 3

Generally, the attendees of the SMART presentations and webinars expressed their excitement on the contribution of SMART as a great platform to learn and to share wheelchair education integration experience. Participants reported being pleasantly surprised by the quality and the volume of resources available on this early version of SMART. The most prominent changes occurred in appearance and multimedia, structure and design, and navigation. Great efforts were attributed to developing resource search filters given that one of the primary purposes of SMART is to share wheelchair education resources and its ease of use relies on the precision and accuracy of its search function.

### Launch Version of the toolkit

Found at this link: www.smart.wheelchairnetwork.org, SMART is continuously monitored and improved by the ISWP Integration Sub-committee based on the needs and the feedback of past, current and future SMART users. To date, contributions have been received from six educators from MRS and HRS, from occupational therapy, physical therapy, and prosthetics and orthotics, in four languages. The types of resources currently available on SMART include course syllabi, PowerPoint slide decks, laboratory guides, case studies, links to open-source evidence-based resources (e.g., Wheelchair Skills Program), and online interactive modules. In the period of September 17, 2018, and August 13, 2019, there were 1385 SMART users from 86 countries having opened 1326 sessions (total of 13, 278 pageviews).

## Discussion

To the best of our knowledge, SMART is the first and largest collection of open-access wheelchair teaching materials for wheelchair education integration into academic curricula. SMART is a product of the research conducted by the ISWP Integration Sub-committee [14, 18] and the valuable collaborations between ISWP, other organizations, educational institutions, and individual wheelchair service provision educators. Considering that 75.4% (*n* = 57) of educators from LRS, MRS, and HRS use their own original material to teach wheelchair education [14], SMART can serve as a reservoir for all existing and future teaching/integration resources whether or not they are published [23–27]. The development of SMART is timely in that it responds to the need to increase the number of skilled wheelchair service provider personnel, a need that has been identified by several global organizations and initiatives [6, 7, 28].

Given that the development of SMART was anchored in the 5 States of Wheelchair Integration into Academic Curricula (advocacy, planning, course development and delivery, first-time implementation, and improvement), which stems from the Model of Wheelchair Service Provision Education Integration [18], it provides a common language for educators worldwide to discuss and improve the wheelchair service provision education offered in university academic curricula. Moreover, SMART features up-to-date evidence-based wheelchair education and integration resources to support educators’ active efforts in increasing or modifying wheelchair education in academic professional rehabilitation programs. SMART also shares ISWP-developed resources, such as the advocacy toolkit (http://pak.wheelchairnetwork.org/), Integration Snapshots, and original research studies [14–16, 18, 29]. Another advantage of SMART is that its wheelchair education resources are not limited to one specific or broad context. Using the extensive search filters, educators may find materials most relevant to their contexts, but also resources useful for global health initiatives should they choose such endeavors. Because SMART and its resources are open-source, there are conditions of use that protect the authorships and ensure that they are used as intended by the authors who shared them.

SMART also faces certain challenges. Given that one of its primary purposes is sharing of education resources, it relies heavily on users’ contributions. Based on the current lack of contributions from LRS, SMART requires more promotion in order to reach any and all educators who may be interested in wheelchair education. At present, our strategy is to actively promote SMART via ISWP and other global organizations, such as the World Federation of Occupational Therapists [30]. Continued efforts by such organizations, and others with special focus on international development, would be of great benefit in reaching educators globally. Finally, as a website, SMART requires regular maintenance and updating, which could be a challenge for ISWP, a non-profit organization. However, as a priority to ISWP, efforts are continually invested to include this endeavor in funding requests and work plans.

### Strengths and limitations of the study

The use of the PAR approach, which facilitated feedback from 115 individuals, ensured that the perspectives and the interests of key stakeholders were well represented and incorporated into SMART. Attempts to globally disseminate SMART included conference presentations in three continents (North America, Africa, and Europe) and webinars in three languages (English, Spanish, and French). The fact that SMART was based on the evidence-based Model of Wheelchair Service Provision Education Integration and its 5 associated States of Wheelchair Integration into Academic Curricula also lends strength to its applicability in all contexts. The development of the SMART may have been limited by reporting bias, in that, meeting, presentation and webinar participants may have felt compelled to provide positive feedback on this initiative. However, given the concrete, constructive feedback that was also received, participant contributions led to modifications that improved the final product.

### Future directions

SMART is a “living document” in that its content will be continuously modified and updated according to the growing evidence in wheelchair-related research and due to the contributions made to SMART. ISWP will maintain oversight of the SMART content, its organization and its functionality. Feedback and suggestions from current and prospective users will be integrated into updates. Several modifications are already planned for the next updates, such as availability in different languages, both for the toolkit itself and for the available resources. A new feature will be developed to help SMART users identify solutions or partners to assist in overcoming barriers. Future studies will evaluate the ease of use and the effectiveness of the toolkit. Should there be the development of a community of practice of wheelchair educators, the effects of SMART must be evaluated as lessons can be learned in how to assist the enhancement of wheelchair service provision education. With the active involvement of educators, students, wheelchair experts, and ISWP, an Educators’ Package will be developed to explicitly guide educators in how to use SMART and other teaching resources in hopes of increasing adoption and integration of wheelchair service provision education into professional rehabilitation academic programs.

## Conclusion

SMART contributes to alleviating the global need for appropriate wheelchair service provision to wheelchair users by facilitating the integration of wheelchair service provision education in professional rehabilitation academic programs in all resource settings. SMART serves not only as a reservoir of educational resources to teach wheelchair education, but also as a community space for people who are invested in training the next generation of wheelchair professionals. SMART is one ever-evolving solution towards better wheelchair service provision worldwide.

## Data Availability

The datasets used and/or analyzed during the current study are available from the corresponding author on reasonable request.

## Abbreviations

CLASP: Consolidating Logistics for Assistive Technology Supply and Provision
ESS: European Seating Symposium
GATE: Global Cooperation on Assistive Technology
GREAT: Global Research, Innovation and Education on Assistive Technology
HRS: High-resourced setting
ISWP: International Society of Wheelchair Professionals
LRS: Low-resourced setting
MRS: Middle-resourced setting
PAR: Participatory action research
RESNA: Rehabilitation Engineering and Assistive Technology Society of North America
SMART: Seating and Mobility Academic Resource Toolkit
WFOT: World Federation of Occupational Therapists
WHO: World Health Organization
